# Genotyping of *Salmonella* Typhi using 8-loci multi locus VNTR analysis

**DOI:** 10.1186/s13099-016-0094-4

**Published:** 2016-04-04

**Authors:** Hongxia Wang, Baowei Diao, Zhigang Cui, Meiying Yan, Biao Kan

**Affiliations:** State Key Laboratory of Infectious Disease Prevention and Control, National Institute for Communicable Disease Control and Prevention, Chinese Center for Disease Control and Prevention, 155, Changbai Road, Changping, Beijing 102206 China; Collaborative Innovation Center for Diagnosis and Treatment of Infectious Diseases, Hangzhou, 310006 China

**Keywords:** *Salmonella* Typhi, MLVA, PFGE

## Abstract

**Background:**

Typhoid fever has caused severe epidemics in many Asian and African countries. The early detection of outbreaks and their sources may promote the prevention and control of typhoid fever, for which effective and timely molecular subtyping techniques are required. Pulsed field gel electrophoresis (PFGE) is routinely used as the molecular typing technique for foodborne and waterborne pathogens. However, maneuverable techniques remain necessary to expedite the experimental procedure and obtain more effective subtyping. The multilocus loci of a variable number of tandem repeats (VNTR) analysis (MLVA) is a polymerase chain reaction (PCR)-based subtyping method.

**Methods:**

MLVA method and PFGE based on *Xba* I enzyme were applied to the 103 *Salmonella* Typhi (*S*. Typhi) isolated from different years and regions. Dendrograms were constructed and analyzed to help understand the data. The Simpson’s index of diversity (*D* value) was calculated to estimate the discriminatory power of MLVA and PFGE. In addition, a set of endogenous 3 bp DNA ladder markers were established to accurately determine the repeat copy number of the VNTR with only a 3 bp repetitive unit, using microfluidics chip-based electrophoresis to generate comparable VNTR data in the public health laboratory network.

**Results:**

The established 8-loci MLVA for *S*. Typhi subtyping had higher discriminatory power than PFGE. In some cases, PFGE could not distinguish the strains isolated over long intervals and with different epidemic provinces. By contrast, 8-loci MLVA distinctly distinguished these strains, and the strains with the same MLVA patterns were from the same or contiguous years and the same province, showing its significance in epidemiological discrimination. The established set of endogenous 3 bp DNA ladder markers improved the accuracy and reproducibility of VNTR analysis using microfluidics chip-based electrophoresis to 100 %.

**Conclusions:**

Eight VNTRs can be used for the MLVA analysis of the 103 *S*. Typhi isolates. MLVA based on the 8-loci had higher discriminatory power than PFGE for *S*. Typhi subtyping. The 8-loci MLVA is easier for the analysis and interpretation of relationships between strains compared to PFGE.

**Electronic supplementary material:**

The online version of this article (doi:10.1186/s13099-016-0094-4) contains supplementary material, which is available to authorized users.

## Background

Typhoid fever is a major public health concern in developing countries. It accounts for an estimated 22 million cases and approximately 1,100,000 deaths annually worldwide [1]. In certain areas of China, the annual incidence rate is approximately 15.3 cases per 100,000 persons, and epidemic or sporadic outbreaks remain common [2].

The prevention and control of the proliferation of typhoid fever rely heavily on the early detection of outbreak and the early identification of its source, for which epidemiology survey only is insufficient due to the long incubation period, sporadic outbreak sites and timing of typhoid. Molecular typing provides useful information about genetic connections among isolates from different sources and times and is ultimately able to identify the source of outbreaks. However, the genetic homogeneity of *S.* Typhi has hampered the application of some molecular typing methods [3–5]. Pulsed-field gel electrophoresis (PFGE) and multilocus variable number of tandem repeats analysis (MLVA) are often used for the molecular typing of *S*. Typhi [6–11], although MLVA and PFGE showed a limited capability to identify epidemiologically linked isolates [10]. PFGE is a routine technique for investigating the outbreaks caused by certain foodborne and waterborne pathogens, including *S*. Typhi [8, 12–15]; however, it is a complicated, time-consuming procedure, and exhibits limited discriminatory power for *S.* Typhi [16]. MLVA is a more maneuverable and rapid procedure with high reproducibility and ease of data sharing; however, it must be standardized. The public health laboratory network requires the standardization of *S.* Typhi MLVA, in which the selection of VNTR loci and the accurate sizing of PCR products are important steps. Ten VNTRs in total reported in the different studies are highly polymorphic in *S.* Typhi [6, 7, 10, 11]. In this study, we evaluated the molecular typing capability of all the reported VNTRs reported in those studies with the 103 *S*. Typhi strains isolated in 1959–2007 and seven typhoid high-incidence provinces in China. For accurate sizing of the PCR products, agarose gel electrophoresis and multicolor capillary electrophoresis using a capillary DNA analyzer and sequencing was used [6, 7, 11, 17]. However, the former has very low resolution and accuracy [6, 7], and the latter two require expensive capillary DNA analyzers and rigorous user training, which hampers its widespread use in public health laboratories. Capillary electrophoresis was also performed on microchip devices, such as an Agilent 2100 Bioanalyzer and QIAxcel System [18–21], which are relatively inexpensive and simple to operate. To achieve widespread use of MLVA in clinical and prefectural public health laboratories, which require standard operation procedures and had microchip devices, we improved the accuracy and reproducibility of VNTR analysis using microfluidics chip-based electrophoresis to generate comparable VNTR data in this study.

## Results and discussions

### Eight VNTRs were identified with the discriminatory ability for MLVA typing of the Chinese epidemic *S*. Typhi strains

We summarized the VNTR loci identified in the previous studies [6, 7, 10, 11] in *S*. Typhi, in which the *D* values were more than 0.1. A total of 10 loci were selected, including Sal02 (Sty37), Sal06 (Sty39), Sal10, Sal11 (TR1 or Sty41), Sal16 (Sty44 or STTR5), Sal20 (Sty40), Sal22 (TR5 or Sty42), TR2 (Sty45), TR4500 (Sty20) and TR4699 (Sty25). We have also searched the tandem repeats with the genome sequences of *S*. Typhi strain CT18 and Ty2 using program VNTRDB, but no new VNTR locus was found. We evaluated the discriminatory power of these loci with 103 *S*. Typhi strains isolated from different years and provinces in China. Nine (Sal02, Sal06, Sal10, Sal11, Sal16, Sal20, Sal22, TR4699 and TR2) were easily amplified by PCR, and only one amplicon was obtained in each reaction. TR4500 was not amplified in 32 strains. The amplicons from 71 TR4500-PCR-positive strains were sequenced. Six strains had a 7 bp deletion downstream of the repeats, four had a 55 bp insertion between the second and third VNTR loci, and one strain contained a 15 bp insertion between the first and second loci (Additional file 1: Tables S4, S5, S6). This locus was excluded in this study because of its complexity, except for the VNTR copy repeating. The locus Sal10 had only one allele in the 103 strains and one contained a 711 bp insertion downstream of the repeat region (Additional file 1: Table S7). This locus was also excluded in this study because no diversity was observed in the 103 test strains. Finally, eight VNTRs (Sal02, Sal06, Sal11, Sal16, Sal20, Sal22, TR4699 and TR2) were selected for the further MLVA analysis of *S*. Typhi. The numbers of repetitions at eight loci in the 103 *S.* Typhi strains are listed in Additional file 1: Table S3, and the characteristics of the eight loci are listed in Table 1. The sizes of the repeat units ranged from 3 to 8 bps. The numbers of alleles varied from 3 to 34, and the alleles contained repeats from 3 to 46. The discriminatory power of each VNTR was calculated. TR2 was the most diverse allele, with a *D* value of 0.965, followed by SAL02, TR4699, Sal11, Sal16, Sal20, Sal06 and Sal22, with a *D* value ranging from 0.182 to 0.925 (Table 1).

**Table 1:** Characteristics of the eight VNTRs observed in the 103 *S*. Typhi strains

VNTR name	Consensus	No. of alleles	Range of no. of repeats	PCR product size range (bp)	*D* values
Sal02	TACCAG	21	6–27	125–251	0.925
Sal06	CTCAAT	4	4–7	168–186	0.416
Sal11(TR1)	AGAAGAA	17	5–20, 30	190–365	0.902
Sal16	ACCATG	13	10– 19, 22, 24, 26	200–296	0.909
Sal20	CAG	9	9, 13–20	172–205	0.795
Sal22	CGTCACG	3	3– 5	163–187	0.182
TR4699	TGTTGG	21	7, 9–22, 24–26, 28, 30, 31	169–313	0.925
TR2	CCAGTTCC	34	4–28, 30–32, 35, 36, 38, 40, 42, 46	297–633	0.965

### MLVA had higher discriminatory power than PFGE

**Fig. 1:**
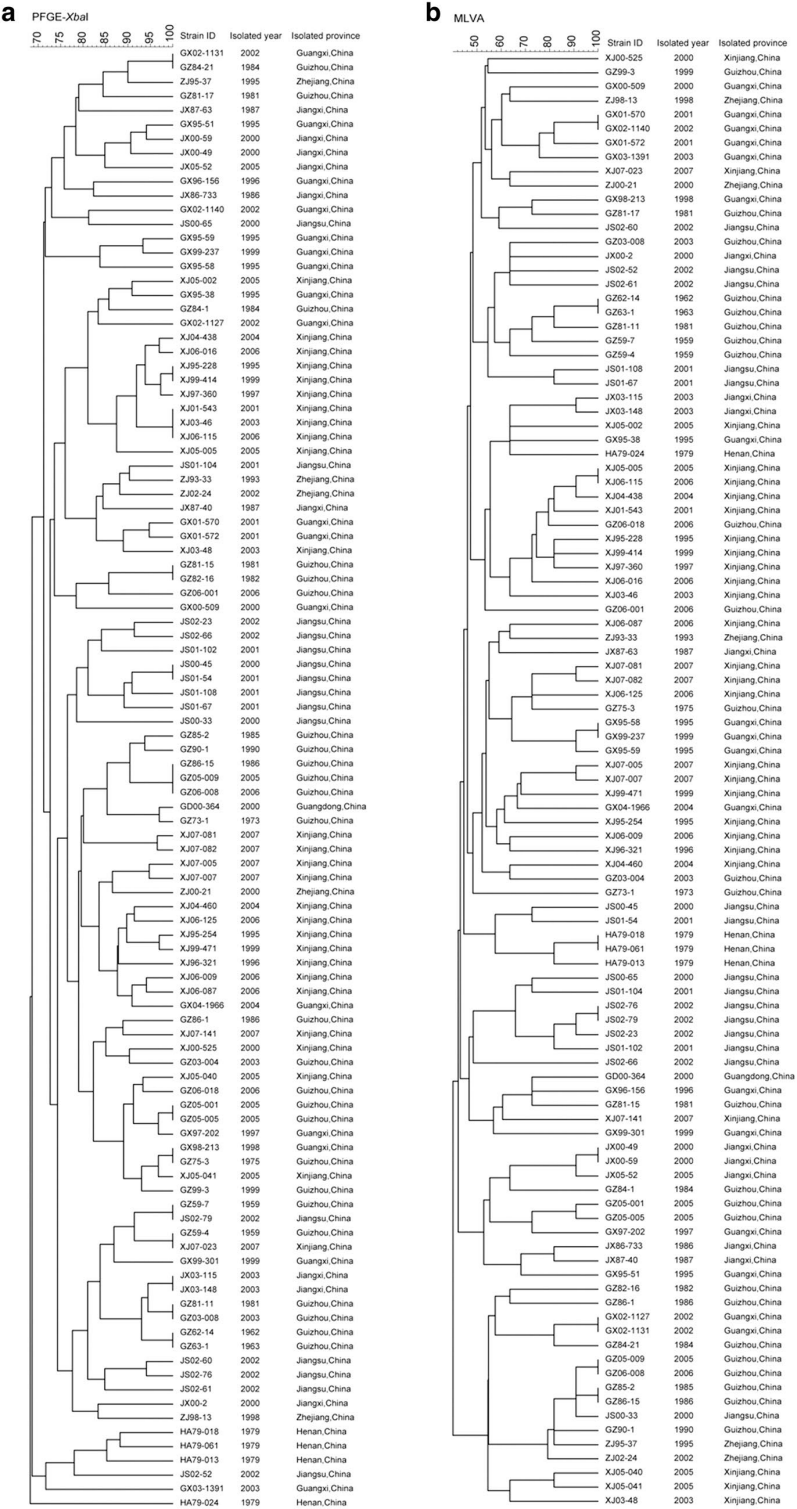
Dendrogram deduced from cluster analysis of the 103 isolates. **a** Cluster analysis of 103 isolates based on PFGE. **b** Cluster analysis of 103 isolates based on MLVA. The dendrogram was evaluated using the Dice coefficient and UPMGA clustering (BioNumerics)

**Fig. 2:**
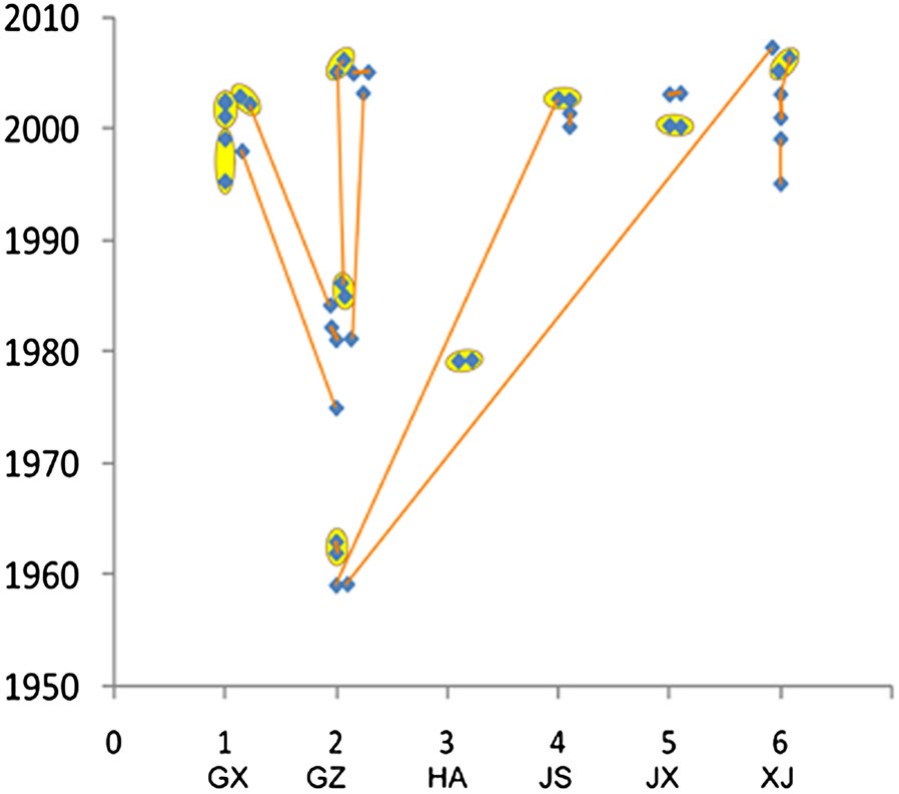
Temporal and spatial distribution of *S*. Typhi isolates showing the same MLVA and PFGE patterns. The *X-axis* represents for different provinces. The *Y-axis* represents different years. *GX* Guangxi, *GZ* Guizhou, *HA* Henan, *JS* Jiangsu, *JX* Jiangxi, *XJ* Xinjiang. The *circle* indicates the same MLVA pattern. The *line* is the same PFGE pattern

In this study, we compared the MLVA method with PFGE for the subtyping of 103 *S*. Typhi strains. PFGE was performed with the *Xba*I enzyme for these strains. MLVA distinguished the 103 strains into 93 profiles, and 10 clusters with the same MLVA pattern were obtained (Figs. 1, 2). All strains sharing the same MLVA pattern were isolated for the same region and the same or contiguous years as follows: HA79-018/HA79-061, JS02-76/JS02-79, JX00-49/JX00-59, and GX02-1127/GX02-1131 were isolated in the same provinces and in the same year, GX01-570/GX02-1140, GZ62-14/GZ63-1, XJ05-005/XJ06-115, GZ05-009/GZ06-008, GZ85-2/GZ86-15 were isolated in the same provinces and 1 year apart, and only GX95-58/GX99-237 was isolated in the same province and at a time period of 4 years (Figs. 1, 2). PFGE distinguished the 103 isolates into 88 profiles, and 13 clusters had the same PFGE pattern in each (Figs. 1, 2). All the strains sharing the same PFGE pattern, except GZ62-14/GZ63-1, were isolated from different regions or more than 18 years apart. GZ62-14/GZ63-1 was isolated in the same provinces and at a period of 1 year. GZ86-15/GZ05-009/GZ06-008 and GZ81-11/GZ03-008 were isolated in the same provinces but at 19, 20 and 22 years, respectively. GX02-1131/GZ84-21 were isolated in neighboring provinces but were separated by 18 years. GX98-213/GZ75-3 were isolated in neighboring provinces but separated by 23 years. GZ59-7/JS02-79 were isolated from distant provinces and separated by 43 years, GZ59-4/XJ07-023 were also isolated from distant provinces and separated by 48 years (Figs. 1, 2).

MLVA displayed a higher *D* value with 0.9981 than PFGE with 0.9968 for the subtyping of these 103 strains. In some cases, PFGE could not distinguish the strains isolated in different epidemic regions, but 8-loci MLVA clearly distinguished the strains from different provinces, which indicated that the discriminatory power of 8-loci MLVA was higher for distinguishing endemic regions than *Xba*I-based PFGE. Most strains with the same PFGE patterns came from 19–43 year intervals or different regions, but the strains with the same MLVA pattern were only isolated in the same or contiguous years and the same province, which indicates that 8-loci MLVA is easier for the analysis and interpretation of the relationships between strains compared to PFGE. Several isolates with the same MLVA pattern were distinguished into different profiles by PFGE. This suggested MLVA also has limited capability in distinguishing some epidemiologically unlinked *S*. Typhi isolates. Either MLVA or PFGE only revealed parts of genomic variance of *S*. Typhi, wheras *S*. Typhi is highly genetically clonal [3–5]. The combination of MLVA and PFGE yielded a higher *D* value of 0.9996 than the two techniques alone and distinguished the 103 isolates into 101 profiles. Strains GZ05-009/GZ06-008 and GZ62-14/GZ63-1 were indistinguishable by both MLVA and PFGE, suggesting a very close genetic relationship in each of these groups.

### The endogenous 3 bp DNA ladder marker effectively enhances the accuracy of copy determination of the VNTR with a short core repeat in the capillary electrophoresis analysis

In MLVA, the accurate copy number identification of the repeat unit in VNTR is critical. Microfluidics chip-based electrophoresis using DNA analyzers such as the Agilent 2100 Bioanalyzer and QIAxcel system achieve accurate DNA fragment length measurement results, including high speed for time savings, ease of use and accuracy. However, it cannot correctly discriminate fragments with length differences of less than 5 bps. In this study, of the eight VNTR loci, Sal20 has the shortest repeat unit with only 3 bp (Table 1), which makes accurate sizing of its PCR product difficult with agarose gel and automatic microfluidics chip-based electrophoresis techniques. In our previous study [22], the accuracy and reproducibility of sizing were very poor using an Agilent 2100 Bioanalyzer and DNA 1000 LabChip Kit. The exogenous internal marker of 80 and 320 bp improved its reproducibility, but the exogenous 20 bp DNA ladder failed to improve its accuracy, particularly for the Sal20 PCR amplicons. Later, the exogenous 3 bp ladder, and internal marker of 145 and 226 bp, amplified from the plasmid pUC19 using primers with a 3 bp gradient, also failed to increase the accuracy of the Sal20 PCR products to 100 % (data not shown). We hypothesize that the sequence and composition of the nucleotide residues of the DNA could affect its migration through the gel during electrophoresis in addition to the length. The endogenous 3 bp DNA ladder and internal marker were prepared from plasmids containing the flanking sequence and repeat unit of the Sal20 locus itself with the serial number of repeat units using the primers for Sal20L/Sal20R. Nine isolates with different numbers of repeat units at the Sal20 locus were selected to evaluate the capability of this set of endogenous DNA ladders and internal markers for accurate sizing (Additional file 1: Table S3). The results showed the reproducibility and accuracy of PCR sizing were increased to 100 % (Table 2). This set of endogenous DNA ladders is also effective for the QIAxcel System (data not shown). The size of all PCR products from 8-loci in the 103 isolates was measured using this set of endogenous DNA ladders and internal markers as well as the microfluidics chip on an Agilent 2100 Bioanalyzer, followed by analysis with the FluorChem SP software. The results were consistent with sequencing results (data not shown). These data indicate that this set of endogenous 3 bp DNA ladder and the internal marker is sufficient for the accurate sizing of PCR products in the MLVA of *S.* Typhi.

**Table 2:** Size of PCR products at Sal20 locus

Isolate	No. of repeats	Predicted PCR product size (bp)	Sizing of PCR products by Agilent 2100 bioanalyzer							
			The low marker and upper marker, ladder included in DNA 1000 LabChip Kit				The new low marker and upper marker, 3 bp ladder			
			Size (bp)			Deviation (bp)	Size (bp)			Deviation (bp)
			First	Second	Third		First	Second	Third	
GZ73-1	9	172	182	181	183	9–11	172	172	172	0
GX97-202	13	184	194	193	195	9–11	184	184	184	0
GZ85-2	14	187	198	196	198	9–11	187	187	187	0
JS01-104	15	190	199	200	202	9–11	190	190	190	0
GZ82-16	16	193	203	202	202	9–11	193	193	193	0
GZ81-15	17	196	206	205	206	9–10	196	196	196	0
GZ84-1	18	199	209	208	210	9–11	199	199	199	0
GX98-213	19	202	212	211	213	9–11	202	202	202	0
ZJ00-21	20	205	216	215	217	10–12	205	205	205	0

In summary, we estimated the molecular subtyping ability of the VNTRs and MLVA for *S*. Typhi. Eight VNTR loci were selected, and this protocol has higher discriminatory power than PFGE (*Xba*I digestion); in particular, MLVA attained more acceptable clustering of the epidemiological information. Although PFGE is widely used to subtype bacterial pathogens, microbial genome re-sequencing is under review for its use in epidemiological investigations, both are time-consuming and multiplex experimental steps are needed. MLVA is advantageous for its rapidity (less than 3 h) and easy-to-perform. Additionally, it may provide a quick screening when more strains are obtained. Increasing the measurement accuracy of the repeat unit copy numbers is critical for MLVA. Our customized marker strategy developed in this study increased the accuracy of the DNA analyzer with the microfluidics chip-based electrophoresis technique to less than 1 bp in the VNTR fragment length detection, using the endogenous 3 bp DNA fragment ladders for the locus. It eliminates the DNA sequence difference and enhances the resolution power of capillary electrophoresis. This customized ladder marker can be distributed to laboratories in the surveillance network to ensure quality control in the MLVA analysis within the network. Additionally, it should be noted that MLVA protocol is still needed to be continuously evaluated during its use in the surveillance of typhoid fever and other diseases, since it utilizes only the genomic variance data of the parts of repeat sequences as the subtyping markers.

## Conclusions

Eight VNTRs can be used for the MLVA analysis of the 103 *S*. Typhi isolates. MLVA based on the 8-loci had higher discriminatory power than PFGE for *S*. Typhi subtyping. The 8-loci MLVA is easier for the analysis and interpretation of relationships between strains compared to PFGE. The established endogenous DNA ladder markers can improve the accuracy and reproducibility of VNTR analysis using microfluidics chip-based electrophoresis.

## Methods

### Strains and DNA preparation

A total of 103 *S.* Typhi strains were used in this study; these strains are listed in Fig. 1 and were isolated from blood samples of typhoid patients. *S.* Typhi strain CT18, which was isolated from Vietnam in 1993, was included as a control. DNA was purified using the NucleoSpin^®^ Tissue kit (MACHEREY–NAGEL, Germany) according to the manufacturer’s instructions.

### VNTR amplification

VNTR amplifications were performed as described previously [6, 7, 11]. Briefly, PCR reactions were performed using 10 pairs of primers specific for 10 VNTR loci (Additional file 1: Table S1) and *Taq* DNA polymerase (TaKaRa, China). All PCR products were sequenced and analyzed with an Agilent 2100 Bioanalyzer (Agilent Technologies, USA).

### The endogenous 3 bp DNA ladder and internal marker preparation

Twenty-six oligonucleotides were designed according to the flanking sequence and repeat sequence of the Sal20 locus with 0 repeats, from 2 to 25 repeats, and 27 repeats in *S.* Typhi (Additional file 1: Table S2). All fragments were synthesized and cloned into the pGEM-T Easy Vector. Next, PCR reactions were performed with the primers Sal20 L/Sal20 R (Additional file 1: Table S1) and 26 of the synthesized oligonucleotides as templates. All amplicons were purified with a QIAquick^®^ PCR Purification Kit (Qiagen, German). Each fragment was adjusted to a final concentration of 14 ng/μl. The endogenous 3 bp DNA ladder was prepared by mixing all fragments in an equal volume (Additional file 1: Table S2). The new internal marker was prepared by mixing the lower and upper markers in equal volume (Additional file 1: Table S2).

### Agilent 2100 bioanalyzer analysis

The PCR products were analyzed using the DNA 1000 LabChip Kit (Agilent Technologies, USA). The analysis of PCR products was performed following the manufacturer’s procedure with some modifications. Briefly, the ladder well was loaded with 1 μl DNA ladder and 5 µl internal marker following gel preparation. Eleven sample wells were loaded with 0.5 µl PCR reaction, 0.5 µl new internal marker, and 5 µl internal marker contained in the kit. The last sample well was loaded with 1 μl of the endogenous 3 bp ladder and 5 µl of internal marker contained in the kit. Then, the chip was inserted into the Agilent 2100 Bioanalyzer and performed following the manufacturer’s procedure. Allele sizes were calculated using the DNA ladder included in the kit and the Agilent 2100 Expert Software version B.02.03.SI307 firmware C.01.055 (Agilent Technologies), as well as the endogenous 3 bp ladder and FluorChem SP software (Version 6.0.0.14, Alpha Innotech Corporation). Each analysis was performed three times.

### PFGE

PFGE was performed using the PulseNet USA with *Salmonella* serovar Braenderup H9812 as the marker according to standard operation procedures described previously [23]. Briefly, agarose-embedded DNA was digested with 50 U of *Xba* I (TaKaRa, China) for 2 h in a water bath at 37 °C. The restriction fragments were separated by electrophoresis in 0.5 × Tris-borate-EDTA (TBE) buffer at 14 °C for 18 h using a Chef Mapper electrophoresis system (Bio-Rad, USA), with pulse times of 2.16–63.8 s. The gels were stained with ethidium bromide, and the DNA bands were visualized by UV transillumination.

### Bioinformatic analysis

The statistical analysis was performed with BioNumerics software (Applied Maths, St-Martens-Latern, Belgium) with 1 % tolerance and 0.8 % optimization using Dice coefficients to compare profiles. Dendrograms were constructed by using the unweighted-pair group method with arithmetic means (UPGMA). The Simpson’s index of diversity (*D* value) was calculated using a Plug-in (Simpsons ID), included in the BioNumerics package [24].

### Supporting data

The data sets supporting the results of this article are included within the article and its additional files.

## Additional file

10.1186/s13099-016-0094-4 The characteristics of VNTR loci and primers for amplification of VNTRs. **Table S2.** The endogenous 3bp DNA ladder and internal marker. **Table S3.** Numbers of repetitions at 8 loci in the 103 *S*. Typhi strains. **Table S4.** 7 bp deletion in TR4500. **Table S5.** 15 bp insertion in TR4500. **Table S6.** 55 bp insertion in TR4500. **Table S7.** 711 bp insertion in Sal10.

## Abbreviations

PFGE: pulsed field gel electrophoresis
VNTR: variable number of tandem repeats
MLVA: variable number of tandem repeats analysis
*S*. Typhi: *Salmonella* Typhi
PCR: polymerase chain reaction
*D* value: the Simpson’s index of diversity
TBE: tris–borate-EDTA
UPGMA: unweighted-pair group method with arithmetic means
