# Supplementary material for: Genotyping of Salmonella Typhi using 8-loci multi locus VNTR analysis
Source: Gut Pathog. 2016 Apr 4;8:14. doi: 10.1186/s13099-016-0094-4 (PMC4819281; doi:10.1186/s13099-016-0094-4)
Supplement: Supplementary file 1 — 10.1186/s13099-016-0094-4 The characteristics of VNTR loci and primers for amplification of VNTRs. Table S2. The endogenous 3bp DNA ladder and internal marker. Table S3. Numbers of repetitions at 8 loci in the 103 S. Typhi strains. Table S4. 7 bp deletion in TR4500. Table S5. 15 bp insertion in TR4500. Table S6. 55 bp insertion in TR4500. Table S7. 711 bp insertion in Sal10. [file 13099_2016_94_MOESM1_ESM.docx]

**Table S1.** The characteristics of VNTR loci and primers for amplification of VNTRs

| Primer | Sequence (5’ to 3’) | Length of repeat unit | Product size (bp) in CT18 (copy no.) | Reference |
| --- | --- | --- | --- | --- |
| Sal02 L | GGAAAGACTGGCGAACAAAT | 6 bp | 149 (10) | 26 |
| Sal02 R | TCGCCAATACCATGAGTACG |  |  |  |
| Sal06 L | TTGGTCGCGGAACTATAACTG | 6 bp | 174 (5) | 26 |
| Sal06 R | CTTCGTCTGATTGCCACTCC |  |  |  |
| Sal10 L | AAGCGACGTTCTTCTGCAAC | 12 bp | 196 (2) | 26 |
| Sal10 R | TGGAATATGATGGCATGACG |  |  |  |
| Sal11(TR1) L | GCCAACGATCGCTACTTTTT | 7 bp | 239 (12) | 26 |
| Sal11(TR1) R | GCGCATACTACACCGATCAC |  |  |  |
| Sal16 L | CCATGGCTGCAGTTAATTTCT | 6 bp | 224 (14) | 26 |
| Sal16 R | TGATACGCTTTTGACGTTGC |  |  |  |
| Sal20 L | CAGCCGACACAACTTAACGA | 3 bp | 193 (16) | 26 |
| Sal20 R | ACTGTACCGTGCGCGTTT |  |  |  |
| Sal22 L | GCCAGAGGGTTCATTTTCAA | 7 bp | 184 (6) | 26 |
| Sal22 R | ATGCGACGCCGTTTTACTAC |  |  |  |
| TR2U | CCCTGTTTT TCGTGCTGATACG | 8bp | 511(28) | 7 |
| TR2L | CAGAGGATATCGCAACAATCGG |  |  |  |
| TR4500U | CGTTGCTGCTCCGAAAT | 6bp | 366(4) | 11 |
| TR4500L | GCGGTGAAGTGGAAAAAG |  |  |  |
| TR4699U | TATTCTACTTCAGTCCCCCC | 6bp | 247(17) | 11 |
| TR4699L | AACCTCCCTGTATCTACCAA |  |  |  |

**Table S2.** The endogenous 3bp DNA ladder and internal marker

| Numbers of repetitions | Fragment length(bp) | Nucleotide sequence |
| --- | --- | --- |
| 3 bp ladder | | |
| 2 | 151 | CAGCCGACACAACTTAACGAAGTGCCGTGGAACGAGCAGACGCCAGAACAACGCCAGCAAACGCTA*CAGCGC*TGGGCTCAAACGCAGCCTGTTCAGCAACCACGCACGCAGCCGCGGGTAAACGAACAGCCGCAAACGCGCACGGTACAGT |
| 3 | 154 | CAGCCGACACAACTTAACGAAGTGCCGTGGAACGAGCAGACGCCAGAACAACGCCAGCAAACGCTA*CAGCGCCAGT*GGGCTCAAACGCAGCCTGTTCAGCAACCACGCACGCAGCCGCGGGTAAACGAACAGCCGCAAACGCGCACGGTACAGT |
| 4 | 157 | CAGCCGACACAACTTAACGAAGTGCCGTGGAACGAGCAGACGCCAGAACAACGCCAGCAAACGCTA*CAGCGCCAGCGT*TGGGCTCAAACGCAGCCTGTTCAGCAACCACGCACGCAGCCGCGGGTAAACGAACAGCCGCAAACGCGCACGGTACAGT |
| 5 | 160 | CAGCCGACACAACTTAACGAAGTGCCGTGGAACGAGCAGACGCCAGAACAACGCCAGCAAACGCTA*CAGCGCCAGCGTCAG*TGGGCTCAAACGCAGCCTGTTCAGCAACCACGCACGCAGCCGCGGGTAAACGAACAGCCGCAAACGCGCACGGTACAGT |
| 6 | 163 | CAGCCGACACAACTTAACGAAGTGCCGTGGAACGAGCAGACGCCAGAACAACGCCAGCAAACGCTA*CAGCGCCAGCGTCAGGCG*TGGGCTCAAACGCAGCCTGTTCAGCAACCACGCACGCAGCCGCGGGTAAACGAACAGCCGCAAACGCGCACGGTACAGT |
| 7 | 166 | CAGCCGACACAACTTAACGAAGTGCCGTGGAACGAGCAGACGCCAGAACAACGCCAGCAAACGCTA*CAGCGCCAGCGTCAGGCGCAG*TGGGCTCAAACGCAGCCTGTTCAGCAACCACGCACGCAGCCGCGGGTAAACGAACAGCCGCAAACGCGCACGGTACAGT |
| 8 | 169 | CAGCCGACACAACTTAACGAAGTGCCGTGGAACGAGCAGACGCCAGAACAACGCCAGCAAACGCTA*CAGCGCCAGCGTCAGGCGCAGCAG*TGGGCTCAAACGCAGCCTGTTCAGCAACCACGCACGCAGCCGCGGGTAAACGAACAGCCGCAAACGCGCACGGTACAGT |
| 9 | 172 | CAGCCGACACAACTTAACGAAGTGCCGTGGAACGAGCAGACGCCAGAACAACGCCAGCAAACGCTA*CAGCGCCAGCGTCAGGCGCAGCAGCAG*TGGGCTCAAACGCAGCCTGTTCAGCAACCACGCACGCAGCCGCGGGTAAACGAACAGCCGCAAACGCGCACGGTACAGT |
| 10 | 175 | CAGCCGACACAACTTAACGAAGTGCCGTGGAACGAGCAGACGCCAGAACAACGCCAGCAAACGCTA*CAGCGCCAGCGTCAGGCGCAGCAGCAGCAG*TGGGCTCAAACGCAGCCTGTTCAGCAACCACGCACGCAGCCGCGGGTAAACGAACAGCCGCAAACGCGCACGGTACAGT |
| 11 | 178 | CAGCCGACACAACTTAACGAAGTGCCGTGGAACGAGCAGACGCCAGAACAACGCCAGCAAACGCTA*CAGCGCCAGCGTCAGGCGCAGCAGCAGCAGCAG*TGGGCTCAAACGCAGCCTGTTCAGCAACCACGCACGCAGCCGCGGGTAAACGAACAGCCGCAAACGCGCACGGTACAGT |
| 12 | 181 | CAGCCGACACAACTTAACGAAGTGCCGTGGAACGAGCAGACGCCAGAACAACGCCAGCAAACGCTA*CAGCGCCAGCGTCAGGCGCAGCAGCAGCAGCAGCAG*TGGGCTCAAACGCAGCCTGTTCAGCAACCACGCACGCAGCCGCGGGTAAACGAACAGCCGCAAACGCGCACGGTACAGT |
| 13 | 184 | CAGCCGACACAACTTAACGAAGTGCCGTGGAACGAGCAGACGCCAGAACAACGCCAGCAAACGCTA*CAGCGCCAGCGTCAGGCGCAGCAGCAGCAGCAGCAGCAG*TGGGCTCAAACGCAGCCTGTTCAGCAACCACGCACGCAGCCGCGGGTAAACGAACAGCCGCAAACGCGCACGGTACAGT |
| 14 | 187 | CAGCCGACACAACTTAACGAAGTGCCGTGGAACGAGCAGACGCCAGAACAACGCCAGCAAACGCTA*CAGCGCCAGCGTCAGGCGCAGCAGCAGCAGCAGCAGCAGCAG*TGGGCTCAAACGCAGCCTGTTCAGCAACCACGCACGCAGCCGCGGGTAAACGAACAGCCGCAAACGCGCACGGTACAGT |
| 15 | 190 | CAGCCGACACAACTTAACGAAGTGCCGTGGAACGAGCAGACGCCAGAACAACGCCAGCAAACGCTA*CAGCGCCAGCGTCAGGCGCAGCAGCAGCAGCAGCAGCAGCAGCAG*TGGGCTCAAACGCAGCCTGTTCAGCAACCACGCACGCAGCCGCGGGTAAACGAACAGCCGCAAACGCGCACGGTACAGT |
| 16 | 193 | CAGCCGACACAACTTAACGAAGTGCCGTGGAACGAGCAGACGCCAGAACAACGCCAGCAAACGCTA*CAGCGCCAGCGTCAGGCGCAGCAGCAGCAGCAGCAGCAGCAGCAGCAG*TGGGCTCAAACGCAGCCTGTTCAGCAACCACGCACGCAGCCGCGGGTAAACGAACAGCCGCAAACGCGCACGGTACAGT |
| 17 | 196 | CAGCCGACACAACTTAACGAAGTGCCGTGGAACGAGCAGACGCCAGAACAACGCCAGCAAACGCTA*CAGCGCCAGCGTCAGGCGCAGCAGCAGCAGCAGCAGCAGCAGCAGCAGCAG*TGGGCTCAAACGCAGCCTGTTCAGCAACCACGCACGCAGCCGCGGGTAAACGAACAGCCGCAAACGCGCACGGTACAGT |
| 18 | 199 | CAGCCGACACAACTTAACGAAGTGCCGTGGAACGAGCAGACGCCAGAACAACGCCAGCAAACGCTA*CAGCGCCAGCGTCAGGCGCAGCAGCAGCAGCAGCAGCAGCAGCAGCAGCAGCAG*TGGGCTCAAACGCAGCCTGTTCAGCAACCACGCACGCAGCCGCGGGTAAACGAACAGCCGCAAACGCGCACGGTACAGT |
| 19 | 202 | CAGCCGACACAACTTAACGAAGTGCCGTGGAACGAGCAGACGCCAGAACAACGCCAGCAAACGCTA*CAGCGCCAGCGTCAGGCGCAGCAGCAGCAGCAGCAGCAGCAGCAGCAGCAGCAGCAG*TGGGCTCAAACGCAGCCTGTTCAGCAACCACGCACGCAGCCGCGGGTAAACGAACAGCCGCAAACGCGCACGGTACAGT |
| 20 | 205 | CAGCCGACACAACTTAACGAAGTGCCGTGGAACGAGCAGACGCCAGAACAACGCCAGCAAACGCTA*CAGCGCCAGCGTCAGGCGCAGCAGCAGCAGCAGCAGCAGCAGCAGCAGCAGCAGCAGCAG*TGGGCTCAAACGCAGCCTGTTCAGCAACCACGCACGCAGCCGCGGGTAAACGAACAGCCGCAAACGCGCACGGTACAGT |
| 21 | 208 | CAGCCGACACAACTTAACGAAGTGCCGTGGAACGAGCAGACGCCAGAACAACGCCAGCAAACGCTA*CAGCGCCAGCGTCAGGCGCAGCAGCAGCAGCAGCAGCAGCAGCAGCAGCAGCAGCAGCAGCAG*TGGGCTCAAACGCAGCCTGTTCAGCAACCACGCACGCAGCCGCGGGTAAACGAACAGCCGCAAACGCGCACGGTACAGT |
| 22 | 211 | CAGCCGACACAACTTAACGAAGTGCCGTGGAACGAGCAGACGCCAGAACAACGCCAGCAAACGCTA*CAGCGCCAGCGTCAGGCGCAGCAGCAGCAGCAGCAGCAGCAGCAGCAGCAGCAGCAGCAGCAGCAG*TGGGCTCAAACGCAGCCTGTTCAGCAACCACGCACGCAGCCGCGGGTAAACGAACAGCCGCAAACGCGCACGGTACAGT |
| 23 | 214 | CAGCCGACACAACTTAACGAAGTGCCGTGGAACGAGCAGACGCCAGAACAACGCCAGCAAACGCTA*CAGCGCCAGCGTCAGGCGCAGCAGCAGCAGCAGCAGCAGCAGCAGCAGCAGCAGCAGCAGCAGCAGCAG*TGGGCTCAAACGCAGCCTGTTCAGCAACCACGCACGCAGCCGCGGGTAAACGAACAGCCGCAAACGCGCACGGTACAGT |
| 24 | 217 | CAGCCGACACAACTTAACGAAGTGCCGTGGAACGAGCAGACGCCAGAACAACGCCAGCAAACGCTA*CAGCGCCAGCGTCAGGCGCAGCAGCAGCAGCAGCAGCAGCAGCAGCAGCAGCAGCAGCAGCAGCAGCAGCAG*TGGGCTCAAACGCAGCCTGTTCAGCAACCACGCACGCAGCCGCGGGTAAACGAACAGCCGCAAACGCGCACGGTACAGT |
| 25 | 220 | CAGCCGACACAACTTAACGAAGTGCCGTGGAACGAGCAGACGCCAGAACAACGCCAGCAAACGCTA*CAGCGCCAGCGTCAGGCGCAGCAGCAGCAGCAGCAGCAGCAGCAGCAGCAGCAGCAGCAGCAGCAGCAGCAGCAG*TGGGCTCAAACGCAGCCTGTTCAGCAACCACGCACGCAGCCGCGGGTAAACGAACAGCCGCAAACGCGCACGGTACAGT |
| Low marker | | |
| 0 | 145 | CAGCCGACACAACTTAACGAAGTGCCGTGGAACGAGCAGACGCCAGAACAACGCCAGCAAACGCTATGGGCTCAAACGCAGCCTGTTCAGCAACCACGCACGCAGCCGCGGGTAAACGAACAGCCGCAAACGCGCACGGTACAGT |
| Upper marker | | |
| 27 | 226 | CAGCCGACACAACTTAACGAAGTGCCGTGGAACGAGCAGACGCCAGAACAACGCCAGCAAACGCTA*CAGCGCCAGCGTCAGGCGCAGCAGCAGCAGCAGCAGCAGCAGCAGCAGCAGCAGCAGCAGCAGCAGCAGCAGCAGCAGCAG*TGGGCTCAAACGCAGCCTGTTCAGCAACCACGCACGCAGCCGCGGGTAAACGAACAGCCGCAAACGCGCACGGTACAGT |

**Table S3.** Numbers of repetitions at 8 loci in the 103 *S.*Typhi strains

| Isolate | No. of repetitions at the following locus | | | | | | | |
| --- | --- | --- | --- | --- | --- | --- | --- | --- |
|  | Sal02 | Sal06 | Sal11 | Sal16 | Sal20 | Sal22 | TR4699 | TR2 |
| GZ59-4 | 27 | 5 | 12 | 18 | 18 | 4 | 10 | 12 |
| GZ59-7 | 24 | 5 | 10 | 18 | 18 | 4 | 12 | 13 |
| GZ62-14 | 14 | 5 | 11 | 18 | 18 | 4 | 12 | 30 |
| GZ63-1 | 14 | 5 | 11 | 18 | 18 | 4 | 12 | 30 |
| GZ73-1 | 7 | 5 | 13 | 12 | 9 | 4 | 17 | 5 |
| GZ75-3 | 12 | 5 | 8 | 13 | 14 | 4 | 16 | 46 |
| HA79-013 | 12 | 4 | 16 | 13 | 19 | 4 | 7 | 6 |
| HA79-018 | 14 | 4 | 16 | 13 | 17 | 4 | 7 | 6 |
| HA79-024 | 13 | 5 | 10 | 17 | 17 | 4 | 7 | 4 |
| HA79-061 | 14 | 4 | 16 | 13 | 17 | 4 | 7 | 6 |
| GZ81-11 | 19 | 5 | 11 | 18 | 18 | 4 | 12 | 23 |
| GZ81-15 | 15 | 6 | 12 | 22 | 17 | 4 | 7 | 40 |
| GZ81-17 | 8 | 5 | 12 | 18 | 16 | 4 | 7 | 18 |
| GZ82-16 | 22 | 6 | 10 | 11 | 16 | 4 | 12 | 14 |
| GZ84-1 | 13 | 5 | 14 | 14 | 18 | 4 | 17 | 23 |
| GZ84-21 | 17 | 6 | 11 | 11 | 14 | 4 | 20 | 13 |
| GZ85-2 | 13 | 6 | 10 | 12 | 14 | 4 | 16 | 15 |
| GZ86-1 | 16 | 6 | 10 | 11 | 13 | 4 | 21 | 21 |
| GZ86-15 | 13 | 6 | 10 | 12 | 14 | 4 | 16 | 15 |
| JX86-733 | 9 | 5 | 9 | 14 | 16 | 5 | 18 | 14 |
| JX87-40 | 9 | 5 | 11 | 14 | 16 | 5 | 18 | 15 |
| JX87-63 | 24 | 5 | 8 | 11 | 9 | 4 | 24 | 4 |
| GZ90-1 | 15 | 6 | 10 | 12 | 14 | 4 | 16 | 14 |
| ZJ93-33 | 14 | 5 | 20 | 13 | 17 | 4 | 24 | 42 |
| GX95-38 | 26 | 5 | 10 | 13 | 17 | 4 | 9 | 11 |
| GX95-51 | 12 | 5 | 11 | 14 | 16 | 5 | 16 | 19 |
| GX95-58 | 10 | 5 | 13 | 13 | 14 | 4 | 19 | 13 |
| GX95-59 | 10 | 5 | 13 | 13 | 14 | 4 | 19 | 15 |
| ZJ95-37 | 12 | 6 | 10 | 12 | 14 | 4 | 16 | 17 |
| XJ95-228 | 22 | 5 | 5 | 17 | 17 | 4 | 17 | 31 |
| XJ95-254 | 9 | 5 | 14 | 10 | 14 | 4 | 22 | 15 |
| GX96-156 | 10 | 6 | 12 | 15 | 17 | 4 | 7 | 19 |
| XJ96-321 | 17 | 5 | 14 | 12 | 14 | 4 | 14 | 30 |
| GX97-202 | 12 | 5 | 8 | 14 | 13 | 4 | 12 | 22 |
| XJ97-360 | 22 | 5 | 5 | 17 | 17 | 4 | 18 | 23 |
| GX98-213 | 9 | 5 | 13 | 18 | 19 | 4 | 7 | 18 |
| ZJ98-13 | 16 | 5 | 12 | 16 | 19 | 4 | 10 | 21 |
| GZ99-3 | 21 | 5 | 9 | 10 | 16 | 4 | 19 | 25 |
| XJ99-414 | 22 | 5 | 5 | 17 | 17 | 4 | 20 | 26 |
| XJ99-471 | 15 | 5 | 16 | 10 | 14 | 4 | 15 | 16 |
| GX99-237 | 10 | 5 | 13 | 13 | 14 | 4 | 19 | 13 |
| GX99-301 | 16 | 7 | 6 | 26 | 17 | 4 | 7 | 21 |
| ZJ00-21 | 18 | 5 | 11 | 22 | 20 | 4 | 7 | 11 |
| GX00-509 | 16 | 5 | 11 | 16 | 16 | 4 | 17 | 11 |
| GD00-364 | 16 | 6 | 12 | 24 | 17 | 4 | 7 | 12 |
| XJ00-525 | 17 | 5 | 15 | 17 | 16 | 4 | 28 | 10 |
| JX00-2 | 25 | 5 | 9 | 19 | 18 | 4 | 10 | 25 |
| JX00-49 | 12 | 5 | 10 | 14 | 18 | 5 | 17 | 10 |
| JX00-59 | 12 | 5 | 10 | 14 | 18 | 5 | 17 | 10 |
| JS00-33 | 13 | 6 | 10 | 12 | 14 | 4 | 16 | 6 |
| JS00-45 | 14 | 5 | 16 | 13 | 16 | 4 | 26 | 11 |
| JS00-65 | 12 | 5 | 8 | 15 | 16 | 4 | 7 | 13 |
| JS01-54 | 13 | 5 | 16 | 13 | 16 | 4 | 31 | 28 |
| JS01-67 | 11 | 5 | 30 | 13 | 18 | 4 | 28 | 18 |
| JS01-102 | 14 | 5 | 8 | 15 | 17 | 3 | 7 | 24 |
| JS01-104 | 12 | 5 | 7 | 15 | 15 | 4 | 7 | 11 |
| JS01-108 | 11 | 5 | 30 | 14 | 18 | 4 | 28 | 19 |
| GX01-570 | 18 | 5 | 12 | 16 | 16 | 4 | 16 | 27 |
| GX01-572 | 22 | 5 | 12 | 17 | 16 | 4 | 16 | 27 |
| XJ01-543 | 21 | 5 | 5 | 17 | 17 | 4 | 19 | 19 |
| ZJ02-24 | 13 | 6 | 10 | 12 | 14 | 4 | 13 | 19 |
| GX02-1127 | 18 | 6 | 11 | 11 | 14 | 4 | 21 | 13 |
| GX02-1131 | 18 | 6 | 11 | 11 | 14 | 4 | 21 | 13 |
| GX02-1140 | 18 | 5 | 12 | 16 | 16 | 4 | 16 | 27 |
| JS02-60 | 14 | 7 | 7 | 16 | 16 | 4 | 7 | 18 |
| JS02-61 | 12 | 5 | 11 | 19 | 18 | 4 | 9 | 8 |
| JS02-79 | 12 | 5 | 8 | 15 | 17 | 3 | 7 | 23 |
| JS02-23 | 12 | 5 | 8 | 15 | 17 | 3 | 7 | 15 |
| JS02-52 | 12 | 5 | 9 | 16 | 18 | 4 | 13 | 12 |
| JS02-66 | 25 | 5 | 10 | 15 | 20 | 4 | 11 | 35 |
| JS02-76 | 12 | 5 | 8 | 15 | 17 | 3 | 7 | 23 |
| JX03-115 | 12 | 5 | 10 | 19 | 17 | 4 | 12 | 16 |
| JX03-148 | 12 | 5 | 10 | 19 | 17 | 4 | 12 | 18 |
| XJ03-46 | 21 | 5 | 5 | 18 | 17 | 4 | 14 | 36 |
| XJ03-48 | 9 | 6 | 15 | 11 | 14 | 4 | 15 | 9 |
| GZ03-004 | 14 | 5 | 13 | 11 | 14 | 4 | 30 | 20 |
| GZ03-008 | 21 | 5 | 12 | 19 | 18 | 4 | 13 | 15 |
| GX03-1391 | 18 | 5 | 6 | 16 | 16 | 4 | 19 | 27 |
| XJ04-438 | 17 | 5 | 5 | 17 | 17 | 4 | 13 | 19 |
| XJ04-460 | 14 | 7 | 18 | 11 | 14 | 4 | 15 | 6 |
| GZ05-001 | 12 | 5 | 9 | 14 | 13 | 4 | 11 | 27 |
| GZ05-005 | 12 | 5 | 9 | 14 | 13 | 4 | 11 | 28 |
| GZ05-009 | 13 | 6 | 10 | 12 | 14 | 4 | 16 | 16 |
| GX04-1966 | 15 | 5 | 11 | 14 | 14 | 4 | 21 | 7 |
| XJ05-002 | 18 | 5 | 10 | 16 | 17 | 4 | 17 | 8 |
| XJ05-005 | 22 | 5 | 5 | 17 | 17 | 4 | 13 | 19 |
| XJ05-040 | 19 | 6 | 18 | 10 | 14 | 4 | 15 | 21 |
| XJ05-041 | 19 | 6 | 19 | 10 | 14 | 4 | 15 | 22 |
| JX05-52 | 12 | 5 | 10 | 14 | 18 | 5 | 17 | 27 |
| GZ06-001 | 21 | 6 | 13 | 17 | 17 | 4 | 12 | 38 |
| GZ06-008 | 13 | 6 | 10 | 12 | 14 | 4 | 16 | 16 |
| GZ06-018 | 16 | 5 | 5 | 17 | 13 | 4 | 13 | 19 |
| XJ06-009 | 15 | 5 | 17 | 11 | 14 | 4 | 14 | 4 |
| XJ06-016 | 23 | 5 | 5 | 17 | 17 | 4 | 19 | 35 |
| XJ06-087 | 14 | 5 | 8 | 10 | 14 | 4 | 24 | 8 |
| XJ06-115 | 22 | 5 | 5 | 17 | 17 | 4 | 13 | 19 |
| XJ06-125 | 7 | 5 | 8 | 13 | 14 | 4 | 28 | 31 |
| XJ07-005 | 15 | 5 | 11 | 10 | 14 | 4 | 11 | 32 |
| XJ07-007 | 16 | 5 | 11 | 10 | 14 | 4 | 11 | 32 |
| XJ07-023 | 18 | 5 | 11 | 18 | 16 | 4 | 14 | 14 |
| XJ07-081 | 6 | 5 | 8 | 13 | 14 | 4 | 25 | 25 |
| XJ07-082 | 6 | 5 | 8 | 13 | 14 | 4 | 25 | 15 |
| XJ07-141 | 16 | 6 | 13 | 15 | 14 | 4 | 7 | 16 |

**Table S4.** 7 bp deletion in TR4500

Alignment Report of '7 bp deletion in TR4500' - ClustalW (Slow/Accurate, IUB)

Majority TTGCGGTGAAGTGAAAAAGGTTCGTAAAGCCGTTGACGTTCAGGAGGCGGCAGCCGACCAGGGCGCTTGCGCCCCTTGGA

---------+---------+---------+---------+---------+---------+---------+---------+

10 20 30 40 50 60 70 80

---------+---------+---------+---------+---------+---------+---------+---------+

TR4500 in CT18.seq -TGCGGTGAAGTGAAAAAGGTTCGTAAAGCCGTTGACGTTCAGGAGGCGGCAGCCGACCAGGGCGCTTGCGCCCCTTGGA 79

TR4500 in XJ01-543.seq TTGCGGTGAAGTGAAAAAGGTTCGTAAAGCCGTTGACGTTCAGGAGGCGGCAGCCGACCAGGGCGCTTGCGCCCCTTGGA 80

TR4500 in XJ05-005.seq TGGCGGTGAAGTGAAAAAGGTTCGTAAAGCCGTTGACGTTCAGGAGGCGGCAGCCGACCAGGGCGCTTGCGCCCCTTGGA 80

TR4500 in XJ06-016.seq TGGCGGTGAAGTGAAAAAGGTTCGTAAAGCCGTTGACGTTCAGGAGGCGGCAGCCGACCAGGGCGCTTGCGCCCCTTGGA 80

TR4500 in XJ95-228.seq --GCGGTGAAGTGAAAAAGGTTCGTAAAGCCGTTGACGTTCAGGAGGCGGCAGCCGACCAGGGCGCTTGCGCCCCTTGGA 78

TR4500 in XJ97-360.seq TGGCGGTGAAGTGAAAAAGGTTCGTAAAGCCGTTGACGTTCAGGAGGCGGCAGCCGACCAGGGCGCTTGCGCCCCTTGGA 80

TR4500 in XJ99-414.seq TTGCGGTGAAGTGAAAAAGGTTCGTAAAGCCGTTGACGTTCAGGAGGCGGCAGCCGACCAGGGCGCTTGCGCCCCTTGGA 80

Majority CTC-------------------TAACTGTCCCCCTGTTGAAAATCTGAACAAATCAGGGGGTGATTTACCCGATATTAAA

---------+---------+---------+---------+---------+---------+---------+---------+

90 100 110 120 130 140 150 160

---------+---------+---------+---------+---------+---------+---------+---------+

TR4500 in CT18.seq CTCGGACTCGGACTCGTGGCAATAACTGTCCCCCTGTTGAAAATCTGAACAAATCAGGGGGTGATTTACCCGATATTAAA 159

TR4500 in XJ01-543.seq CTC-------------------TAACTGTCCCCCTGTTGAAAATCTGAACAAATCAGGGGGTGATTTACCCGATATTAAA 141

TR4500 in XJ05-005.seq CTC-------------------TAACTGTCCCCCTGTTGAAAATCTGAACAAATCAGGGGGTGATTTACCCGATATTAAA 141

TR4500 in XJ06-016.seq CTC-------------------TAACTGTCCCCCTGTTGAAAATCTGAACAAATCAGGGGGTGATTTACCCGATATTAAA 141

TR4500 in XJ95-228.seq CTC-------------------TAACTGTCCCCCTGTTGAAAATCTGAACAAATCAGGGGGTGATTTACCCGATATTAAA 139

TR4500 in XJ97-360.seq CTC-------------------TAACTGTCCCCCTGTTGAAAATCTGAACAAATCAGGGGGTGATTTACCCGATATTAAA 141

TR4500 in XJ99-414.seq CTC-------------------TAACTGTCCCCCTGTTGAAAATCTGAACAAATCAGGGGGTGATTTACCCGATATTAAA 141

Majority ACCATGAATGAGAAGGAACTGCAGGATTATCTCCACAATATGGGCCAGAAGGAACGGCGGGAACTTACAGCCAGGTTGAG

---------+---------+---------+---------+---------+---------+---------+---------+

170 180 190 200 210 220 230 240

---------+---------+---------+---------+---------+---------+---------+---------+

TR4500 in CT18.seq ACCATGAATGAGAAGGAACTGCAGGATTATCTCCACAATATGGGCCAGAAGGAACGGCGGGAACTTACAGCCAGGTTGAG 239

TR4500 in XJ01-543.seq ACCATGAATGAGAAGGAACTGCAGGATTATCTCCACAATATGGGCCAGAAGGAACGGCGGGAACTTACAGCCAGGTTGAG 221

TR4500 in XJ05-005.seq ACCATGAATGAGAAGGAACTGCAGGATTATCTCCACAATATGGGCCAGAAGGAACGGCGGGAACTTACAGCCAGGTTGAG 221

TR4500 in XJ06-016.seq ACCATGAATGAGAAGGAACTGCAGGATTATCTCCACAATATGGGCCAGAAGGAACGGCGGGAACTTACAGCCAGGTTGAG 221

TR4500 in XJ95-228.seq ACCATGAATGAGAAGGAACTGCAGGATTATCTCCACAATATGGGCCAGAAGGAACGGCGGGAACTTACAGCCAGGTTGAG 219

TR4500 in XJ97-360.seq ACCATGAATGAGAAGGAACTGCAGGATTATCTCCACAATATGGGCCAGAAGGAACGGCGGGAACTTACAGCCAGGTTGAG 221

TR4500 in XJ99-414.seq ACCATGAATGAGAAGGAACTGCAGGATTATCTCCACAATATGGGCCAGAAGGAACGGCGGGAACTTACAGCCAGGTTGAG 221

Majority ACTGGTAAAAXXXXXXXXXXXXXXXXXXXXXXXXXXXXXXXXXXXXXXXXXXXXXXXX

---------+---------+---------+---------+---------+--------

250 260 270 280 290

---------+---------+---------+---------+---------+--------

TR4500 in CT18.seq ACTGGTAAAA 249

TR4500 in XJ01-543.seq ACTGGTAAAA 231

TR4500 in XJ05-005.seq ACTGGTAAAA 231

TR4500 in XJ06-016.seq ACTGGTAAAA 231

TR4500 in XJ95-228.seq ACTGGTAAAACCGAAGCGGAAAACAGTATACAAACAGAATATTTCGAGCAGCAACGAT 277

TR4500 in XJ97-360.seq ACTGGTAAAACAC 234

TR4500 in XJ99-414.seq ACTGGTAAAACC 233

**Table S5.** 15 bp insertion in TR4500

Alignment Report of '15bp insertion in TR4500' - ClustalW (Slow/Accurate, IUB)

Majority TGCGGTGAATGTGAAAAAGGTTCGTAAAGCCGTTGACGTTCAGGAGGCGGCAGCCGACCAGGGCGCTTGCGCCCCTTGGA

---------+---------+---------+---------+---------+---------+---------+---------+

10 20 30 40 50 60 70 80

---------+---------+---------+---------+---------+---------+---------+---------+

TR4500 in CT18.seq TGCGGTGAA-GTGAAAAAGGTTCGTAAAGCCGTTGACGTTCAGGAGGCGGCAGCCGACCAGGGCGCTTGCGCCCCTTGGA 79

TR4500 in XJ05-002.seq -GCGGTGAATGTGAAAAAGGTTCGTAAAGCCGTTGACGTTCAGGAGGCGGCAGCCGACCAGGGCGCTTGCGCCCCTTGGA 79

Majority CTCGGACTCGTGGCAATAACTGTCGGACTCGGACTCGTGGCAATAACTGTCCCCCTGTTGAAAATCTGAACAAATCAGGG

---------+---------+---------+---------+---------+---------+---------+---------+

90 100 110 120 130 140 150 160

---------+---------+---------+---------+---------+---------+---------+---------+

TR4500 in CT18.seq CTCGGACTC---------------GGACTC------GTGGCAATAACTGTCCCCCTGTTGAAAATCTGAACAAATCAGGG 138

TR4500 in XJ05-002.seq CTCGGACTCGTGGCAATAACTGTCGGACTCGGACTCGTGGCAATAACTGTCCCCCTGTTGAAAATCTGAACAAATCAGGG 159

Majority GGTGATTTACCCGATATTAAAACCATGAATGAGAAGGAACTGCAGGATTATCTCCACAATATGGGCCAGAAGGAACGGCG

---------+---------+---------+---------+---------+---------+---------+---------+

170 180 190 200 210 220 230 240

---------+---------+---------+---------+---------+---------+---------+---------+

TR4500 in CT18.seq GGTGATTTACCCGATATTAAAACCATGAATGAGAAGGAACTGCAGGATTATCTCCACAATATGGGCCAGAAGGAACGGCG 218

TR4500 in XJ05-002.seq GGTGATTTACCCGATATTAAAACCATGAATGAGAAGGAACTGCAGGATTATCTCCACAATATGGGCCAGAAGGAACGGCG 239

Majority GGAACTTACAGCCAGGTTGAGACTGGTAAAAXX

---------+---------+---------+---

250 260 270

---------+---------+---------+---

TR4500 in CT18.seq GGAACTTACAGCCAGGTTGAGACTGGTAAAA 249

TR4500 in XJ05-002.seq GGAACTTACAGCCAGGTTGAGACTGGTAAAACC

**Table S6.** 55 bp insertion in TR4500

Alignment Report of '55 bp insertion in TR4500' - ClustalV (Weighted)

Majority T---GCGGTGAACGTG-AAAAAGGTTCGTAAAGCCGTTGACGTTCAGGAGGCGGCAGCCGACCAGGGCGCTTGCGCCCCT

---------+---------+---------+---------+---------+---------+---------+---------+

10 20 30 40 50 60 70 80

---------+---------+---------+---------+---------+---------+---------+---------+

TR4500 in CT18.seq T---GCGGTGAA-GTG-AAAAAGGTTCGTAAAGCCGTTGACGTTCAGGAGGCGGCAGCCGACCAGGGCGCTTGCGCCCCT 75

TR4500 in GX00-509.seq TT--GCGGTGAACGTG-AAAAAGGTTCGTAAAGCCGTTGACGTTCAGGAGGCGGCAGCCGACCAGGGCGCTTGCGCCCCT 77

TR4500 in GX01-570.seq TATGGCGGTGAAC-TG-AAAAAGGTTCGTAAAGCCGTTGACGTTCAGGAGGCGGCAGCCGACCAGGGCGCTTGCGCCCCT 78

TR4500 in GX01-572.seq T------------GTG-AAAAAGGTTCGTAAAGCCGTTGACGTTCAGGAGGCGGCAGCCGACCAGGGCGCTTGCGCCCCT 67

TR4500 in GX02-1140.seq T-TATCGGTGAAAGGGGAAAAAGGTTCGTAAAGCCGTTGACGTTCAGGAGGCGGCAGCCGACCAGGGCGCTTGCGCCCCT 79

Majority TGGACTCGGTAAAGCCGTTGACGTTCAGGAGGCGGCAGCCGACCAGGGCGCTTGCGCCCCTTGGACTCGGACTCGTGGCA

---------+---------+---------+---------+---------+---------+---------+---------+

90 100 110 120 130 140 150 160

---------+---------+---------+---------+---------+---------+---------+---------+

TR4500 in CT18.seq TGGACTC-------------------------------------------------------GGACTCGGACTCGTGGCA 100

TR4500 in GX00-509.seq TGGACTCGGTAAAGCCGTTGACGTTCAGGAGGCGGCAGCCGACCAGGGCGCTTGCGCCCCTTGGACTCGGACTCGTGGCA 157

TR4500 in GX01-570.seq TGGACTCGGTAAAGCCGTTGACGTTCAGGAGGCGGCAGCCGACCAGGGCGCTTGCGCCCCTTGGACTCGGACTCGTGGCA 158

TR4500 in GX01-572.seq TGGACTCGGTAAAGCCGTTGACGTTCAGGAGGCGGCAGCCGACCAGGGCGCTTGCGCCCCTTGGACTCGGACTCGTGGCA 147

TR4500 in GX02-1140.seq TGGACTCGGTAAAGCCGTTGACGTTCAGGAGGCGGCAGCCGACCAGGGCGCTTGCGCCCCTTGGACTCGGACTCGTGGCA 159

Majority ATAACTGTCCCCCTGTTGAAAATCTGAACAAATCAGGGGGTGATTTACCCGATATTAAAACCATGAATGAGAAGGAACTG

---------+---------+---------+---------+---------+---------+---------+---------+

170 180 190 200 210 220 230 240

---------+---------+---------+---------+---------+---------+---------+---------+

TR4500 in CT18.seq ATAACTGTCCCCCTGTTGAAAATCTGAACAAATCAGGGGGTGATTTACCCGATATTAAAACCATGAATGAGAAGGAACTG 180

TR4500 in GX00-509.seq ATAACTGTCCCCCTGTTGAAAATCTGAACAAATCAGGGGGTGATTTACCCGATATTAAAACCATGAATGAGAAGGAACTG 237

TR4500 in GX01-570.seq ATAACTGTCCCCCTGTTGAAAATCTGAACAAATCAGGGGGTGATTTACCCGATATTAAAACCATGAATGAGAAGGAACTG 238

TR4500 in GX01-572.seq ATAACTGTCCCCCTGTTGAAAATCTGAACAAATCAGGGGGTGATTTACCCGATATTAAAACCATGAATGAGAAGGAACTG 227

TR4500 in GX02-1140.seq ATAACTGTCCCCCTGTTGAAAATCTGAACAAATCAGGGGGTGATTTACCCGATATTAAAACCATGAATGAGAAGGAACTG 239

Majority CAGGATTATCTCCACAATATGGGCCAGAAGGAACGGCGGGAACTTACAGCCAGGTTGAGACTGGTAAAAXX

---------+---------+---------+---------+---------+---------+---------+-

250 260 270 280 290 300 310

---------+---------+---------+---------+---------+---------+---------+-

TR4500 in CT18.seq CAGGATTATCTCCACAATATGGGCCAGAAGGAACGGCGGGAACTTACAGCCAGGTTGAGACTGGTAAAA 249

TR4500 in GX00-509.seq CAGGATTATCTCCACAATATGGGCCAGAAGGAACGGCGGGAACTTACAGCCAGGTTGAGACTGGTAAAAC 307

TR4500 in GX01-570.seq CAGGATTATCTCCACAATATGGGCCAGAAGGAACGGCGGGAACTTACAGCCAGGTTGAGACTGGTAAAACC 309

TR4500 in GX01-572.seq CAGGATTATCTCCACAATATGGGCCAGAAGGAACGGCGGGAACTTACAGCCAGGTTGAGACTGGTAAAAA 297

TR4500 in GX02-1140.seq CAGGATTATCTCCACAATATGGGCCAGAAGGAACGGCGGGAACTTACAGCCAGGTTGAGACTGGTAAA 307

**Table S7.** 711 bp insertion in Sal10

Alignment Report of '711 bp insertion in Sal10' - ClustalW (Slow/Accurate, IUB)

Majority AGCGACGTTCTTCTGCAACGCAGGCAGCGTCAGCGTGTGGGTCATTGAGGACGTGTGATGAGCGTTCCCGGCGGCAGAGC

---------+---------+---------+---------+---------+---------+---------+---------+

10 20 30 40 50 60 70 80

---------+---------+---------+---------+---------+---------+---------+---------+

CT18-Sal10.seq AGCGACGTTCTTCTGCAACGCAGGCAGCGTCAGCGTGTGGGTCATTGAGGACGTGTGATGAGCGTTCCCGGCGGCAGAGC 80

JS02-60-Sal10.seq ---------------------------------CGTGTGGGTCATTGAGGACGTGTGATGAGCGTTCCCGGCGGCAGAGC 47

Majority GCTGCCGGGAAGCGAAATTACGGCAGCGGCGTCGGCAGCGGCGTTTTTTGTCTATGGAAAACCCCCAGCTAGGCTGGGGG

---------+---------+---------+---------+---------+---------+---------+---------+

90 100 110 120 130 140 150 160

---------+---------+---------+---------+---------+---------+---------+---------+

CT18-Sal10.seq GCTGCCGGGAAGCGAAATTACGGCAGCGGCGTCGGCAGCGGCGTTTTTT------------------------------- 129

JS02-60-Sal10.seq GCTGCCGGGAAGCGAAATTACGGCAGCGGCGTCGGCAGCGGCGTTTTTTGTCTATGGAAAACCCCCAGCTAGGCTGGGGG 127

Majority TTCCGGAAAGCTTTCAGCTTTAAGCCAGTTATTAAAACCCCTTTTGATTTGTTAAAACATCTTGCGGTCTGGCGACTGCA

---------+---------+---------+---------+---------+---------+---------+---------+

170 180 190 200 210 220 230 240

---------+---------+---------+---------+---------+---------+---------+---------+

CT18-Sal10.seq -------------------------------------------------------------------------------- 129

JS02-60-Sal10.seq TTCCGGAAAGCTTTCAGCTTTAAGCCAGTTATTAAAACCCCTTTTGATTTGTTAAAACATCTTGCGGTCTGGCGACTGCA 207

Majority AAAGTTCAACAAGAAATCAAAAGGGGGTCCCAATGGGGGACGAAAAGAGCTTAGCGCACACCCGATGGAACTGTAAATAT

---------+---------+---------+---------+---------+---------+---------+---------+

250 260 270 280 290 300 310 320

---------+---------+---------+---------+---------+---------+---------+---------+

CT18-Sal10.seq -------------------------------------------------------------------------------- 129

JS02-60-Sal10.seq AAAGTTCAACAAGAAATCAAAAGGGGGTCCCAATGGGGGACGAAAAGAGCTTAGCGCACACCCGATGGAACTGTAAATAT 287

Majority CACATAGTTTTCGCGCCCAAATACCGAAGACAAGCGTTCTATGGAGAGAAGCGTAGGGCAGTAGGCAGCATATTAAGAAA

---------+---------+---------+---------+---------+---------+---------+---------+

330 340 350 360 370 380 390 400

---------+---------+---------+---------+---------+---------+---------+---------+

CT18-Sal10.seq -------------------------------------------------------------------------------- 129

JS02-60-Sal10.seq CACATAGTTTTCGCGCCCAAATACCGAAGACAAGCGTTCTATGGAGAGAAGCGTAGGGCAGTAGGCAGCATATTAAGAAA 367

Majority ATTGTGTGAATGGAAAAACGTACGAATTCTGGAAGCGGAATGTTGTGCAGATCATATTCACATGCTTCTGGAGATCCCGC

---------+---------+---------+---------+---------+---------+---------+---------+

410 420 430 440 450 460 470 480

---------+---------+---------+---------+---------+---------+---------+---------+

CT18-Sal10.seq -------------------------------------------------------------------------------- 129

JS02-60-Sal10.seq ATTGTGTGAATGGAAAAACGTACGAATTCTGGAAGCGGAATGTTGTGCAGATCATATTCACATGCTTCTGGAGATCCCGC 447

Majority CGAAGATGAGTGTGTCGAGTTTCATGGGATATCTGAAGGGTAAAAGTAGTCTGATGCTTTACGAGCAGTTTGGGGATCTA

---------+---------+---------+---------+---------+---------+---------+---------+

490 500 510 520 530 540 550 560

---------+---------+---------+---------+---------+---------+---------+---------+

CT18-Sal10.seq -------------------------------------------------------------------------------- 129

JS02-60-Sal10.seq CGAAGATGAGTGTGTCGAGTTTCATGGGATATCTGAAGGGTAAAAGTAGTCTGATGCTTTACGAGCAGTTTGGGGATCTA 527

Majority AAATTCAAATACAGGAACAGGGAGTTCTGGTGCAGAGGGTACTATGTCGATACGGTGGGTAAGAATACGGCGAAGATACA

---------+---------+---------+---------+---------+---------+---------+---------+

570 580 590 600 610 620 630 640

---------+---------+---------+---------+---------+---------+---------+---------+

CT18-Sal10.seq -------------------------------------------------------------------------------- 129

JS02-60-Sal10.seq AAATTCAAATACAGGAACAGGGAGTTCTGGTGCAGAGGGTACTATGTCGATACGGTGGGTAAGAATACGGCGAAGATACA 607

Majority GGACTACATAAAGCACCAGCTTGAAGAGGATAAAATGGGTGAGCAATTATCGATCCCGTATCCGGGCAGCCCGTTTACGG

---------+---------+---------+---------+---------+---------+---------+---------+

650 660 670 680 690 700 710 720

---------+---------+---------+---------+---------+---------+---------+---------+

CT18-Sal10.seq -------------------------------------------------------------------------------- 129

JS02-60-Sal10.seq GGACTACATAAAGCACCAGCTTGAAGAGGATAAAATGGGTGAGCAATTATCGATCCCGTATCCGGGCAGCCCGTTTACGG 687

Majority GCCGTAAGTAACGAAGTTTGATGCAAATGTCAGATCGTATGCGCCTGTTAGGGCGCGGCTGGTAAGAGAGCCTTACAGGC

---------+---------+---------+---------+---------+---------+---------+---------+

730 740 750 760 770 780 790 800

---------+---------+---------+---------+---------+---------+---------+---------+

CT18-Sal10.seq -------------------------------------------------------------------------------- 129

JS02-60-Sal10.seq GCCGTAAGTAACGAAGTTTGATGCAAATGTCAGATCGTATGCGCCTGTTAGGGCGCGGCTGGTAAGAGAGCCTTACAGGC 767

Majority GCATCAGAAAAACCTCCGGCTATGCCGGAGGATATTTATTCTAACGCGGCGGCGCACTCCGGGTTGGXXXXXXXXXXXXX

---------+---------+---------+---------+---------+---------+---------+---------+

810 820 830 840 850 860 870 880

---------+---------+---------+---------+---------+---------+---------+---------+

CT18-Sal10.seq ----------------------------------------CTAACGCGGCGGCGCACTCCGGCGTCGGACGAGCGACCCG 169

JS02-60-Sal10.seq GCATCAGAAAAACCTCCGGCTATGCCGGAGGATATTTATTCTAACGCGGCGGCGCACTCCGCGTCGA 834

Majority XXXXXXXXXXXXXXXXXXXXXXXXXXX

---------+---------+-------

890 900

---------+---------+-------

CT18-Sal10.seq TTGCAGCGTCATGCCATCATATTCCAA 196

JS02-60-Sal10.seq 834
